# Quantitative Proteomic Analysis of Cell Cycle of the Dinoflagellate *Prorocentrum donghaiense* (Dinophyceae)

**DOI:** 10.1371/journal.pone.0063659

**Published:** 2013-05-15

**Authors:** Da-Zhi Wang, Ying-Jiao Zhang, Shu-Fei Zhang, Lin Lin, Hua-Sheng Hong

**Affiliations:** State Key Laboratory of Marine Environmental Science/College of the Environment and Ecology, Xiamen University, Xiamen, China; University of Connecticut, United States of America

## Abstract

Dinoflagellates are the major causative agents of harmful algal blooms in the coastal zone, which has resulted in adverse effects on the marine ecosystem and public health, and has become a global concern. Knowledge of cell cycle regulation in proliferating cells is essential for understanding bloom dynamics, and so this study compared the protein profiles of *Prorocentrum donghaiense* at different cell cycle phases and identified differentially expressed proteins using 2-D fluorescence difference gel electrophoresis combined with MALDI-TOF-TOF mass spectrometry. The results showed that the synchronized cells of *P. donghaiense* completed a cell cycle within 24 hours and cell division was phased with the diurnal cycle. Comparison of the protein profiles at four cell cycle phases (G1, S, early and late G2/M) showed that 53 protein spots altered significantly in abundance. Among them, 41 were identified to be involved in a variety of biological processes, e.g. cell cycle and division, RNA metabolism, protein and amino acid metabolism, energy and carbon metabolism, oxidation-reduction processes, and ABC transport. The periodic expression of these proteins was critical to maintain the proper order and function of the cell cycle. This study, to our knowledge, for the first time revealed the major biological processes occurring at different cell cycle phases which provided new insights into the mechanisms regulating the cell cycle and growth of dinoflagellates.

## Introduction

Dinoflagellates are not only the primary producers in marine and fresh water ecosystems, but they are also the major causative agents of harmful algal blooms (HABs) in the coastal zone [1], [2]. Moreover, many of them are able to produce various toxins that impact human health through the consumption of sea foods contaminated by the toxic dinoflagellates, or through water or aerosol exposure [3]. In addition to these adverse impacts, dinoflagellate toxins are responsible for the death of marine fish, shellfish, mammals, birds, and other animals depending on the marine food web [4]–[7]. In the past few decades, the frequency, intensity and geographic distribution of dinoflagellate causing HABs have increased significantly and so have attracted considerable public concern. Many studies have been devoted to the physical, chemical, and biological mechanisms involved in HABs [8]. However, little is known concerning the molecular mechanisms regulating the formation of HABs.

The growth of a marine phytoplankton population results directly from the completion of a cell cycle and, therefore, study of cell cycle progression and its regulation might help to reveal the mechanisms underlying the growth and bloom formation of dinoflagellates. In eukaryotic cells, the cell cycle consists of G1, S, G2 and M phases, and cell cycle progression is regulated by both cyclins and cyclin-dependent kinases (CDKs). Their interactions drive the cell through the different stages of the cell cycle and subsequently regulate cell growth [9]. Dinoflagellates follow a typical eukaryotic G1-S-G2-M cell cycle [10] and a few cyclin and CDK–like proteins or genes have been found in dinoflagellates. A proliferating cell nuclear antigen (PCNA) gene has been identified in many dinoflagellate species [11]–[16], and its expression is high in the late night and early day in *Pyrocystis lunula* [11], while reaches the highest in the S phase in *Karenia brevis* [15], [17]. A mitotic cyclin gene has also been detected in *Gonyaulax polyedra* and *Alexandrium fundyense* with high expression in the G2/M-phase cells [18], [19]. A cyclin B-like protein in *K. brevis* may regulate the cell cycle proceeding from the G2 to the M phase [20]. CDC2 like-kinase is also detected in *Gambierdiscus toxicus* and its expression is observed through the cell cycle, but only presents activity in the late phase of the dark cycle [21]. Some eukaryotic cell cycle regulation factors, i.e. CDK and histone kinase activity, are also reported in other HAB species [22], [23]. These studies suggest that dinoflagellates might follow the same cell cycle regulation mechanism as other eukaryotic organisms, with cyclins and CDKs playing important roles in the cell cycle regulation and population generation of dinoflagellates. However, so far, little is known about the cell cycle progression and its regulation of dinoflagellates due to their unique features, such as extranuclear spindles, lack of nucleosomes, enormous genomes in liquid crystal states, and permanently condensed chromosomes throughout the cell cycle [24], [25]. These unusual features provide challenges to the study of dinoflagellates when using the traditional biochemical methods and molecular technologies, and this results in the global lack of dinoflagellate genomic information, and seriously impedes our understanding of cell growth regulation and the blooming mechanisms of dinoflagellates [26].

*Prorocentrum donghaiense* is one of the key dinoflagellate species which causes extensive blooms along the coast of China, and results in serious damage to the ecosystem and mariculture as well as a threat to public health [27]. Much effort has been devoted to *in situ* investigations of environmental conditions with the focus on physical, chemical, and biological proxies during the course of *P.* *donghaiense* blooms to understand the mechanisms controlling the occurrence and maintenance of these blooms at the population level [27], [28]. Other mechanisms, occurring at the molecular and cellular levels that regulate cell division and growth, are poorly investigated.

Proteins are the “workhorse” molecules of life, taking part in essentially every structure and activity of life, i.e. cell growth, proliferation and homeostasis. Thus, studying cell cycle-dependent protein expression in a global manner should help to uncover the cell cycle regulation of dinoflagellates. Two dimensional difference gel electrophoresis (2-D DIGE) is a high resolution gel-based quantitative proteomic method widely used to detect protein expressing alterations after treatment [29]. This technology not only facilitates the quantification over a comparatively wide dynamic range with high accuracy, but also enables relative quantification with reference to an internal standard, thereby also facilitating the analysis of an adequate set of biological replicates in order to obtain the most significant data on protein regulation. This technique has recently been applied to dinoflagellate study [30].

In this study, we used the quantitative proteomic approach, 2-D DIGE, to compare the protein profiles of *P. donghaiense* collected at different cell cycle phases, and identified differentially expressed proteins using MALDI-TOF-TOF mass spectrometry. The purpose of this study was to mining proteins participating in cell cycle progression, and hence to reveal the molecular processes involved in the cell cycle regulation of dinoflagellates at the proteome level.

## Results

### Cell Growth Dynamics of *P. donghaiense*

**Figure 1:**
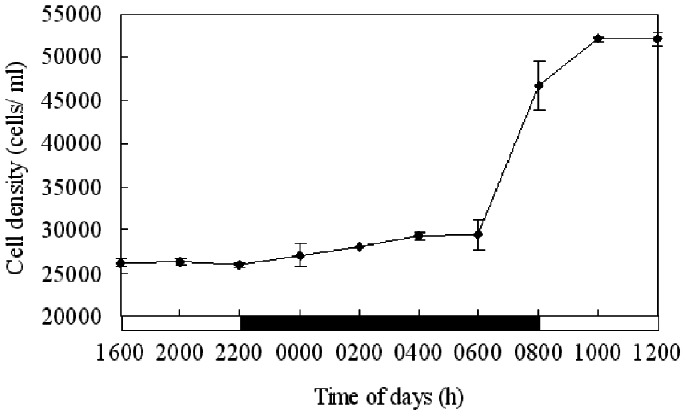
Variation of *P.* donghaiense cell density over time. The cell cycle experiment started at 1600 of the second day and ended at 1200 of the third day. Cell division was phased with the diurnal cycle, and cell density was maintained relatively stable until 0600 h and increased sharply between 0600 and 1000 h, mainly from 0600 to 0800 h.

Starting from a cell density of approximately 13, 000 cells mL^−1^ at 1600 h on the first day, the initial culture grew rapidly. The cell density reached approximately 26, 000 cells mL^−1^ at 1600 h on the second day. From 2000 h on the second day to 1200 h on the third day, the cell density was counted every two hours (Figure 1). Cell density was maintained relatively stable until 0600 h on the third day. Then it increased sharply in the next two hours and reached its maximum at 1000 h, indicating cell division was phased with the diurnal cycle and occurred between 0600 and 1000 h, mainly from 0600 to 0800 h. At other times of day, the cell density varied only a little.

### Cell Cycle Phases of *P. donghaiense*

**Figure 2:**
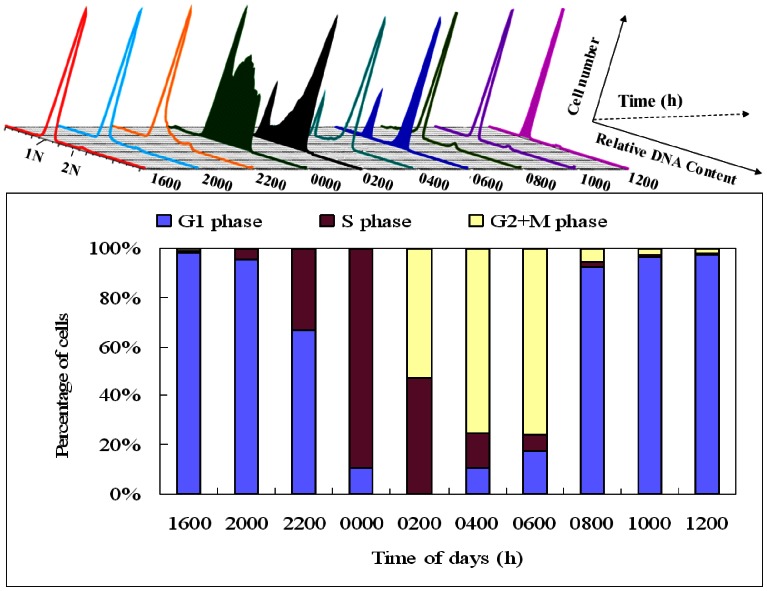
Diel phasing of the cell cycle in *P.* donghaiense determined using flow cytometry. (A) Flow cytograms of PI-stained synchronous *P. donghaiense* cells were plotted, with the x axis representing the relative DNA amount, and the y axis representing the cell number. (B) The percentage of cells at different cell cycle phases during a cell cycle. The majority of cells stayed in G1 phase from 0800 to 2200 h, S phase from 2200 to 0200 h, and G2/M phase from 0200 to 0800 h.

Cells of *P. donghaiense* were harvested every two hours throughout one diel cycle. Each time point was performed in triplicate and FACS analyses were carried out in parallel (Figure 2). The majority of cells stayed in the G1 phase from 0800 to 2200 h, S phase from 2200 to 0200 h, and G2/M phase from 0200 h to 0800 h (Figure 2). At 0000 h, the cell cycle phase distributions of cells in the G1, G2/M, and S phases were 11%, 0% and 89%, respectively. At 0200 h, the cell cycle phase distribution of cells in G1, G2/M and S phase were 0%, 53% and 47%, respectively. At 0600 h, the cell cycle phase distributions of cells in the G1, G2/M and S phase were 18%, 76% and 6%, respectively. At 1200 h, the cell cycle phase distributions of cells in the G1, G2/M, and S phase were 97%, 2% and 1%, respectively. Based on the above results, four samples collected at 0000, 0200, 0600 and 1200 h corresponding to S, early G2/M, late G2/M and G1 phases, were selected for further proteomic analysis.

### 2-D DIGE Analysis of Differential Protein Expression

**Figure 3:**
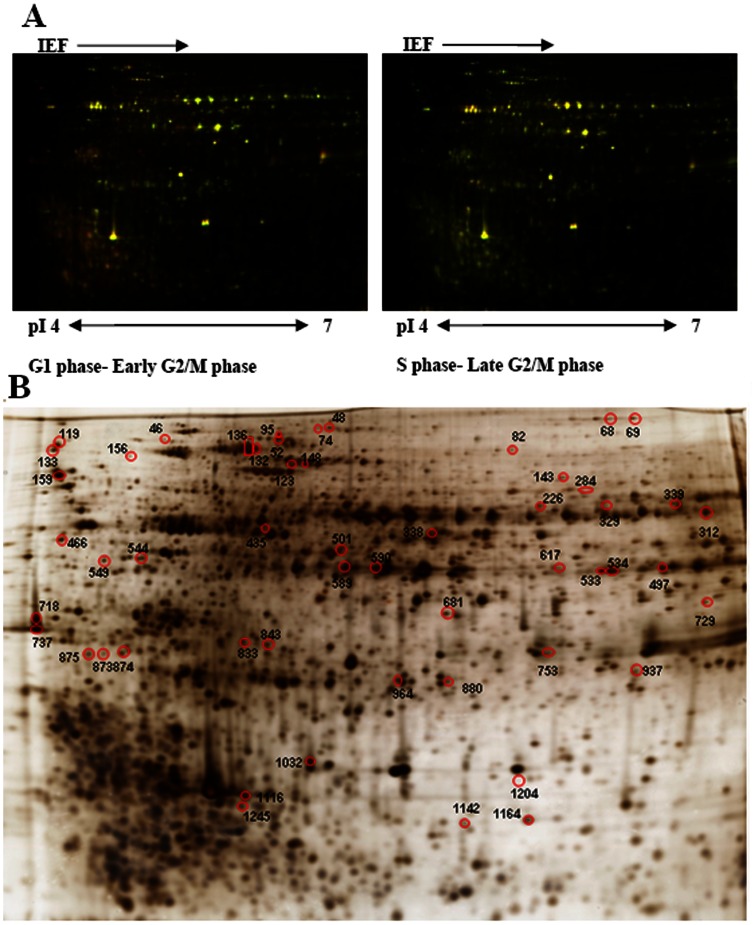
2-D DIGE analysis of *P.* donghaiense at the different cell cycle phases. (A) Representative 2-D DIGE image for protein expression maps using a 12% homogeneous SDS-PAGE gel with the pH range from 4 to 7. (B) Differentially expressed protein spots determined using DeCyder software. Each gel is representative of three independent replicates. 53 protein spots presenting statistically significant alterations in abundance were identified.

To monitor dynamic protein expression, four samples representing the G1, S, early and late G2/M phases were selected for 2-D DIGE analysis. Protein spots were detected automatically using DeCyder 7.0 software. To detect the low abundance proteins, the initial number of protein spots was set up as 2500, and from this initial point, 1123±88 spots (mean ± S.D., n = 18) were detected. Intragel analysis yielded normalized values for each sample with respect to the Cy2 internal standard and direct comparisons between groups. Matching between the different gels was carried out by means of internal standards using the BVA module, and only spots present in 14 of the 18 gels were considered suitable for analysis. Thus, 1020 spots were matched across the different gels, and 53 protein spots presented statistically significant alterations in abundance (ANOVA-1, p≤0.05, and Student’s t-test, p≤0.05) that were greater than 1.5-fold (Figure 3).

### Protein Identification and Variation in Different Cycle Phases

All the protein spots showing alterations were submitted for identification using MALDI-TOF-TOF analysis and then searched against the database using a multilayered strategy for *de novo* protein sequence analysis [31]. 41 protein spots with differential cell cycle expression patterns were identified using MALDI-TOF/TOF MS, while 12 protein spots were not identified. The NCBI ID number, protein name, and average relative change at each time point for identified proteins are listed in Table 1. Detail information including NCBI ID number, protein name, theoretical p*I* value and molecular weight, protein score, as well as protein score C.I. % for identified proteins are listed in Table S1–S4 in Tables S1. Average relative change at each time point for unidentified proteins are listed in Table S5 in Tables S1.

**Table 1:** Differentially expressed proteins of *P. donghaiense* cells in different cell cycle phases.

Spot Id^a^	NCBI^b^	Protein Name	S vs G1		E G2/M vs G1		L G2/M vs G1		1-ANOVA
			ratio	p value	ratio	p valu	ratio	p valu	
Cell cycle and division									
833	190606616	proliferating cell nuclear antigen(PCNA)	1.69	0.092	2.09	0.069	1.3	0.41	0.049
843	190606616	proliferating cell nuclear antigen(PCNA)	3	0.044	2.91	0.047	1.52	0.4	0.029
RNA metabolism									
501	66825125	DEAD/DEAH box helicase domain-containing protein	1.62	0.075	1.67	0.0086	1.43	0.13	0.045
159	312143962	tRNA (5-methylaminomethyl-2-thiouridylate)-methyltransferase	1.55	0.044	1.33	0.13	1.42	0.23	0.14
Protein metabolism/Chaperone									
119	299472226	30S ribosomal protein S1	2.32	0.0037	1.27	–	2.16	0.088	0.019
133	299472226	30S ribosomal protein S1	1.59	0.023	1.15	–	1.48	0.14	0.038
69	294949008	elongation factor 2, putative (EF-2)	2	0.13	–	–	1.85	0.03	0.098
68	193890971	elongation factor 2 (EF-2)	1.96	0.19	–	–	1.78	0.024	0.16
1245	119492760	peptidyl-prolylcis-trans isomerase/cyclophilin, putative (PPIase)	−1.54	0.027	−1.3	0.18	−1.37	0.36	0.54
156	254477818	chaperone protein DnaK (DnaK)	−1.37	0.53	–	–	−3.33	0.044	0.12
136	38885083	heat shock protein 90 (HSP90)	1.55	0.045	1.32	0.23	1.11	0.58	0.076
132	54300510	heat shock protein 90 (HSP90)	1.56	0.019	1.32	0.37	1.23	0.14	0.18
123	294945378	heat shock protein, putative (HSP)	2.01	0.02	1.74	0.012	1.43		0.015
148	294945378	heat shock protein, putative (HSP)	1.87	0.041	−1.57	0.12	1.15	0.51	0.034
874	189425978	chaperonin Cpn10/GroES protein	−2.23	0.033	−1.85	0.032	−1.17	0.29	0.0073
435	227822887	ATP-dependent Clp protease proteolytic subunit 3 (clpP3)	−2.06	0.037	−1.71	–	−1.7	0.048	0.055
Oxidation-reduction process									

Spot ID^a^	NCBI^b^	Protein Name	S vs G1		E G2/M vs G1		L G2/M vs G1		1-ANOVA
			ratio	p value	ratio	p valu	ratio	p valu	
95	160903104	2-oxoglutarate ferredoxinoxidoreductase subunit alpha	−1.24	0.28	−1.38	0.082	−1.8	0.013	0.086
339	146280707	iron-containing alcohol dehydrogenase	−1.09	–	−1.53	–	−1.08	–	0.035
52	260425328	isobutyryl-CoA dehydrogenase	−1.2	0.11	−1.16	0.091	−1.53	0.01	0.019
46	170078578	NAD(P)H-quinone oxidoreductase subunit 4	−1.79	0.019	−1.95	–	−1.4	0.043	0.03
Cell structure and motility									
312	115386236	actin	−1.22	0.22	−1.65	0.077	−1.32	–	0.034
880	159475433	flagellar associated protein	−1.77	0.016	−1.35	0.19	−1.27	0.045	0.055
Energy and Carbon metabolish									
589	35210462	glyceraldehyde-3-phosphate dehydrogenase (GPDH)	2.13	0.016	1.96	0.027	1.42	0.18	0.0089
534	4103875	glyceraldehyde-3-phosphate dehydrogenase (GPDH)	1.84	0.065	1.81	0.051	1.51	0.081	0.041
497	6979054	glyceraldehyde-3-phosphate dehydrogenase (GPDH)	1.62	0.0043	1.57	–	1.33	0.059	0.013
533	4103875	glyceraldehyde-3-phosphate dehydrogenase (GPDH)	1.65	0.055	1.66	0.057	1.52	0.067	0.044
544	99903657	chloroplast sedoheptulose-1,7-bisphosphatase	1.71	0.035	1.63	0.028	1.61	0.027	0.0091
549	99903657	chloroplast sedoheptulose-1,7-bisphosphatase	1.69	0.029	1.6	0.025	1.46	0.043	0.012
590	111608860	fructose 1,6 bisphosphatealdolase 1	1.72	0.043	1.6	0.063	−1.18	0.32	0.023
1032	306430557	light-harvesting protein	−1.53	0.027	−1.31	0.21	−1.42	0.12	0.064
875	58613575	chloroplast light harvesting complex protein	−1.15	0.27	−1.14	0.45	−1.51	0.028	0.09
1116	3355306	light-harvesting polyprotein precursor	−1.19	0.17	−1.46	0.15	−1.58	0.044	0.17
ABC transporters									
1164	289638882	excinuclease ABC, A subunit	−1.67	0.00048	1.38	0.07	−1.53	0.096	0.12
466	56696590	branched-chain amino acid ABC transporter, periplasmic substrate-binding protein	−1.67	0.41	–	–	−2.77	0.035	0.31

Spot ID^a^	NCBI^b^	Protein Name	S vs G1		E G2/M vs G1		L G2/M vs G1		1-ANOVA
			ratio	p value	ratio	p valu	ratio	p valu	
737	259417525	outer membrane porin	3	0.044	2.91	0.047	1.52	0.4	0.029
718	259417525	outer membrane porin	−1.62	0.37	−1.76	0.022	−2.64	0.085	0.35
Amino acid metabolism									
226	296784641	methionine S-adenosyltransferase	1.52	0.055	1.38	0.071	1.26	0.13	0.04
964	211939908	adenosylhomocysteinase	−1.43	0.059	−1.34	0.067	−1.6	0.018	0.049
82	322369524	aspartate/glutamate/uridylate kinase	1.72	0.04	1.5	0.11	1.65	0.064	0.055
Others									
873	117924737	metal dependent phosphohydrolase	−1.69	0.01	−1.46	–	−1.18	0.32	0.066
48	172087756	bifunctional aconitate hydratase 2/2-methylisocitrate dehydratase	1.57	0.033	1.3	0.027	1.37	0.1	0.053

a Spot ID represents the protein spot number on the 2-D DIGE gels.

b Accession numbers according to the NCBI database.

c Spot abundance is expressed as the average ratio of intensities of up-regulated (negative values) or down-regulated (negative values) proteins at S, early G2/M and late G2/M phases compared to G1 phase.

“-”represents no values.

**Figure 4:**
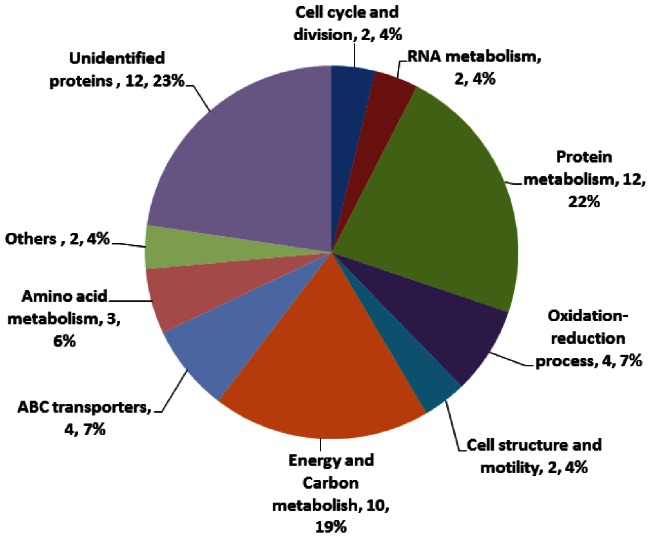
Protein Ontology classification of altered proteins at different cell cycle phases of *P. donghaiense*. The identified proteins were involved in various biological processes, i.e. cell cycle and division, RNA metabolism, protein metabolism, cell structure and motility, energy and carbon metabolism, oxidation-reduction process, amino acid metabolism, ABC transportand other functions.

The identified proteins were involved in various biological processes, i.e. cell cycle and division (4%), RNA metabolism (4%), protein metabolism (22%), cell structure and motility (4%), energy and carbon metabolism (19%), oxidation-reduction process (7%), amino acid metabolism (6%), ABC transport (7%) and other functions (4%), while the unidentified proteins represented 23% of the total proteins (Figure 4). It should be noted that several identified spots represented separable isoforms of the same protein.

Two isoforms of PCNA (spots 833 and 843) were shown to alter in the four cell cycle phases with the lowest level in the G1 phase. In contrast, they exhibited a 2 to 3-fold higher level in the early G2/M and S phases, respectively.Two RNA metabolism proteins, DEAD/DEAH box helicase domain-containing protein (spot 501) and tRNA (5-methylaminomethyl-2-thiouridylate)-methyltransferase (spot 159) were identified. The abundance of the former was enhanced in the S phase while the latter presented higher abundance in the S and early G2/M phases. Two proteins involved in cell structure and motility, actin (spot 312) and flagellar associated protein (spot 880), were identified. The lowest abundance of actin was found in the later G2/M phase, gradually increasing until the G1 phase, then decreasing again. However, the lowest abundance of flagellar associated protein was detected in the S phase, then gradually increasing until the G1 phase. 12 protein spots, identified as protein synthesis, folding and degradation proteins, exhibited significant alterations in abundance during the cell cycle progression, i.e. Rps (spots 119 and 133), EF2 (spots 68 and 69), PPIase (spot 1245), DnaK (spot 156), GroES (spot 874), ClpP3 (spot 435), HSP (spots 123 and 148) and HSP 90 (spots 132 and 136), suggesting that active protein turnover occurred during the cell division cycle. Among them, PPIase, GroES and ClpP3 were depressed as the cells entered the S phase and initial cell division. DnaK was also down-regulated in the S and G2/M phases and presented a very low level in the G2/M phase. However, other proteins were up-regulated as the cells entered the S phase. Three key enzymes involved in glycolytic pathways, i.e. glyceraldehyde-3-phosphate dehydrogenase (GPDH; spots 589, 534, 497 and 533), chloroplast sedoheptulose-1,7- bisphosphatase (spots 544 and 549) and fructose 1,6 bisphosphatealdolase 1 (spot 590) were up-regulated in the S phase, indicating the high energy requirement of the S phase. Three light reaction proteins, including light-harvesting polyprotein precursor (spot 1116), light-harvesting protein (spot 1032) and chloroplast light harvesting complex protein (spot 875), were up-regulated in the G1 phase and down-regulated in other phases, suggesting that active photosynthesis occurred in the G1 phase. Four proteins involved in the oxidative stress response varied with the cell cycle. These were 2-oxoglutarate ferredoxinoxidoreductase subunit alpha (spot 95), isobutyryl-CoA dehydrogenase (spot 52), iron-containing alcohol dehydrogenase (spot 339) and NAD(P)H-quinoneoxidoreductase subunit 4 (spot 46), and they were down-regulated in the S and G2/M phases and up-regulated in the G1 phase. Four proteins were mainly involved in membrane ion transit. They were excinuclease ABC, A subunit (spot 1164), branched-chain amino acid ABC transporter, periplasmic substrate-binding protein (spot 466) and two isoforms of outer membrane porin (spots 737 and 718). Metal dependent phosphohydrolase (spot 873) and bifunctionalaconitatehydratase 2/2-methylisocitrate dehydratase (spot 48) were also identified in this study. These proteins presented differential expression patterns during the cell cycle phases.

### Western Blot Analysis on PCNA

**Figure 5:**
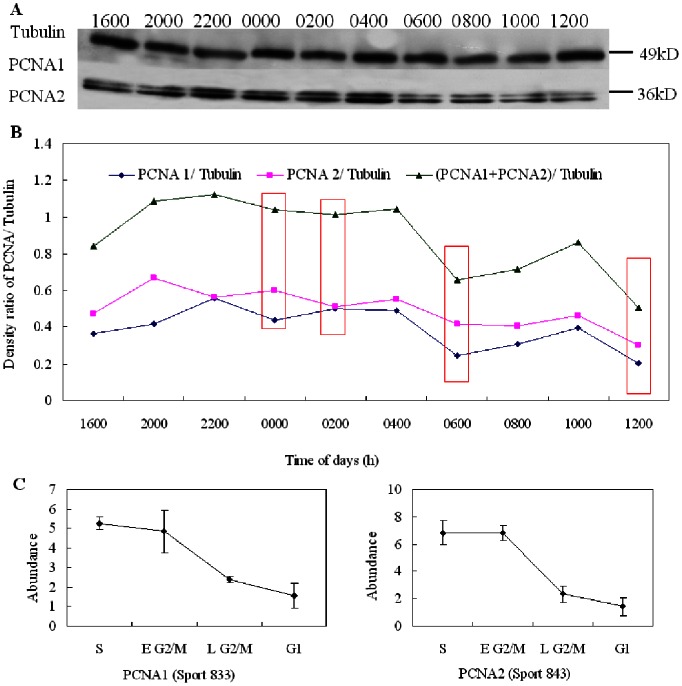
Western blot analysis of PCNA in *P. donghaiense* cells during the cell cycle. Intensities of the proteins were normalized to the corresponding tubulin level. (A) Representative western blots. (B) Western blot results from densitometry analysis. Pink rectangles represent four cell cycle phases. (C) Abundance variations of PCNA1 and PCNA2 in four cell cycle phases in 2-D DIGE gels. The alteration in abundance of PCNA was generally consistent with the variations in 2-D DIGE analysis, which was up-regulated in the S and early G2/M phases and was down-regulated in other cell cycle phases.

Western blot analysis was then conducted to validate PCNA expression during the cell cycle. Results from the western blot showed that the alteration in abundance of PCNA was generally consistent with the variations in 2-D DIGE analysis (Figure 5). PCNA was up-regulated from 2000 to 0400 h, while it was down-regulated at other times, indicating that PCNA played an important role in DNA replication and repair during the S phase of *P. donghaiense*.

## Discussion

The eukaryotic cell division cycle has been studied at the molecular level for decades. It is known that the progression through the cell cycle requires the coordination of several processes including chromosome replication and segregation, as well as cell growth and division, all of which is accomplished by a hierarchical regulatory machinery including biosynthesis, activation, localization and degradation of several key regulators [32]. However, little is known about the dinoflagellate cell cycle. Proteomic analysis is useful in the characterization of the important proteins involved in the bacterial cell cycle [33] and, here, we used the quantitative proteomic approach, 2-D DIGE, to compare the protein profiles of *P. donghaiense* at different cell cycle phases. 41 out of the 53 differentially expressed protein spots involved in a variety of biological processes, e.g. cell cycle and division, RNA metabolism, protein and amino acid metabolism, energy and carbon metabolism, oxidation-reduction processes, and ABC transport, were identified.

PCNA was originally identified as a potential biomarker to study stimulated lymphocytes and other proliferating cells [34]. It is isolated as a protein with elevated level during the S-phase [35]. PCNA plays an essential role in nucleic acid metabolism as a component of the replication and repair of machinery which is synthesized during the cell cycle [36]. PCNA is known as a docking station that encircles DNA and coordinates multiple genetic functions during DNA replication and repair [37], [38]. Because of its association with cell proliferation, PCNA has been widely used for prognosis of tumor and cancer development. These characteristics also make PCNA potentially useful for studying the growth rate of phytoplankton [39]. In dinoflagellates, PCNA is a highly amplified gene, and 41 copies are present in *Pfiesteria piscicida* [40] and 100 copies in *K. brevis* [17], and its expression is found to be slightly higher in the exponential than the stationary growth phase in *P. piscicida* [40]. PCNA gene over-expression occurs in the exponential growth cells of the toxic dinoflagellate *A. catenella* [16], and PCNA transcription per cell decreases obviously from 26.12±3.04 copies in the exponential and transition phases to 5.95±0.79 copies in the stationary phase, and then decreases to below one copy in the declining phase of *P. donghaiense* [15]. In quiescent and senescent cells, there are very low levels of the mRNA and protein of PCNA [41]. In addition, in *K. brevis*, PCNA is unchanged over the cell cycle at the transcriptional level, but is maximally expressed at the S phase [17]. PCNA proteins appear in the late G1 phase of *P. donghaiense*, and gradually increase in abundance and reach a peak in the S phase, and then decline slowly but remain detectable until the G2/M and early M phases [15]. In our study, the abundance of PCNA was higher in the S phase than in the other cell cycle phases, which is consistent with previous studies. These studies indicate that PCNA expression is probably regulated posttranscriptionally in dinoflagellates [17].

The DEAD/DEAH box helicases are a family of proteins involving in various aspects of RNA metabolism, including nuclear transcription, pre-mRNA splicing, ribosome biogenesis, nucleocytoplasmic transport, translation, RNA decay and organellar gene expression [42], [43]. The DEAD box family proteins interact with many cyclin and CDK proteins, which controls cell cycle phase transition [44]–[48]. In our study, the “DEAD/DEAH box helicase domain-containing protein” was identified although the specific function of this protein was not clear. The high expression of this protein in the S and G2/M phases indicated that it might play important roles in RNA metabolism during the cell cycle progression of *P. donghaiense*.

In a typical dinoflagellate cell, two flagella arise from the sides, the transverse flagellum beating sideways around the cell, the longitudinal flagellum beating backwards. This arrangement is known as the dinokont condition [49]. Flagellar structure is required for proper cell division [50]. In the bacterium *Caulobacter crescentus*, a flagellum is built exclusively at the pole that arose from previous cell division. However, we know little about the regulation of flagellum biosynthesis and assembly in dinoflagellates, and it is only deduced that the flagella were re-established in the G1 phase. A network of actin filaments is one of the crucial cytoskeletal structures contributing to the morphological framework of a cell, and which participates in the dynamic regulation of cellular functions. The actin cytoskeleton is re-established after mitosis, allowing cells to regain their extended shape [50]. Our study showed that both flagellar associated protein and actin presented high abundance in the G1 phase and low abundance in the other cell cycle phases, supporting the above observation that cell structure was re-established in the G1 phases immediately after mitosis. However, the exact roles of these two proteins in cell division need further study.

It is estimated that in *C. crescentus* roughly 5% of the proteins are rapidly degraded within one cell cycle equivalent, and more than half are also synthesized in a cell cycle-dependent manner, suggesting that a specific subset of *C. crescentus* proteins is subject to rapid degradation and that proteolysis plays an essential role in *C. crescentus* cell cycle progression and development [33] In our study, we also found a group of proteins involved in protein synthesis, folding and degradation, and most of them were chaperones. Molecular chaperones are a functionally defined set of proteins which assist the structural formation of proteins *in vivo*. Without certain protective mechanisms, such as binding nascent polypeptide chains by molecular chaperones, cellular proteins would lead to misfolding and aggregation. In living organisms, Rps and EF2 are two key proteins participating in protein synthesis [51], PPIase is a modular structure of Tig and mediates ribosome binding [52], [53] and Tig, DnaK, and GroES are three chaperones that participate in the folding of newly synthesized proteins [54]–[56]. In the mammalian system, the molecular chaperones Hsp70 and Hsp90 are involved in the folding and maturation of key regulatory proteins, such as steroid hormone receptors, transcription factors and kinases [57]. HSPs are important factors for progression through certain steps of cell division in both prokaryotic and eukaryotic organisms [58]. CLpP is the proteolytic subunit of the ATP-dependent Clp protease which is encoded by the ClpP genes [59]. ClpP3 cleaves the peptides in various proteins in a process that requires ATP hydrolysis, and thus plays a major role in the degradation of misfolded proteins [60]. Our study found that PPIase, DnaK, GroES and CLpP were up-regulated in the G1 phase, whereas HSP90, 30 Rps and EF-2 were up-regulated in the S phase, suggesting that protein turnover occurred actively during the cell division cycle and these proteins played different roles in maintaining the proper order of the cell cycle. Moreover, the variations of chaperones at different cell cycle phases suggested that chaperones were very important proteins in regulating the cell cycle progression of *P. donghaiense*.

Several proteins involved in the oxidation reduction processes, i.e. 2-oxoglutarate ferredoxin oxidoreductase subunit alpha, isobutyryl-CoA dehydrogenase, iron-containing alcohol dehydrogenase and NAD(P)H-quinone oxidoreductase subunit 4, are also reported to control the cell cycle [61], [62]. Plant cells actively produce reactive oxygen intermediates which are signaling molecules to control processes such as programmed cell death, abiotic stress responses, pathogen defense and systemic signaling [63]. These proteins were depressed in the G2/M phase and were enhanced in the G1 phase in *P. donghaiense*, which could reflect a metabolic peculiarity of the cell type as having a special role in nutrient scavenging. This postulation is in agreement with the observation that the proteolysis enzyme and hydrolases were predominantly synthesized in the G1 phase. All of these enzymes have predicted export signal sequences, implying that they are involved in the degradation of extracellular polypeptides.

*P. donghaiense* is an autotrophic microalgae which uses light as its energy source for cell growth and proliferation. In green plants and algae, light-harvesting proteins act as antennae, capturing photons over a broad frequency spectrum and transferring energy to membrane-bound reaction centers [64]. Various light-harvesting proteins have been reported in dinoflagellates at the transcriptional level [16]. In our study, three light reaction proteins, including light-harvesting polyprotein precursor, light-harvesting protein and chloroplast light harvesting complex protein, were found to be up-regulated in the G1 phase, indicating that active photosynthesis occurred in the G1 phase which provided energy for cellular biosynthesis and metabolism as well as cell division. Furthermore, three proteins involved in glycolytic pathways, i.e. GPDH, chloroplast sedoheptulose-1,7-bisphosphatase and fructose 1,6 bisphosphatealdolase 1, were up-regulated in the S phase, suggesting more energy was produced in the S phase for the synthesis of DNA, a high energy consuming process in this phase. In addition to the glycolytic function, cytosolic GPDH may possess non-glycolytic functions associated with membrane fusion, microtubule bundling, nuclear RNA export, DNA repair, apoptosis, and cell adhesion [65]–[68]. Our study showed that GPDH presented high abundance in the S phase and gradually decreased until the G1 phase, supporting the postulation that GPDH functionally associates with nuclear RNA export and DNA repair during the cell cycle progression.

The ATP-binding cassette (ABC) transporter superfamily is the largest transporter gene family in both prokaryotic and eukaryotic organisms and it transports specific molecules across lipid membranes. These proteins translocate a wide variety of biomolecules including sugars, amino acids, metal ions, peptides, proteins, and a large number of hydrophobic compounds and metabolites across extra- and intracellular membranes [69]. Our study showed that excinuclease ABC, A subunit, branched-chain amino acid ABC transporter and periplasmic substrate-binding protein presented high abundance in the G1 phase, suggesting that active transportation of various biomolecules occurred in the G1 phase. In eukaryotic cells, the G1 phase is characterized by active metabolism, rapid synthesis of RNA and protein, which provides material and energy for subsequent DNA synthesis and cell division. It is interesting that the two isoforms of outer membrane porin identified in this study presented differential expression patterns during the cell cycle: one (spot 718) exhibited high abundance in the G1 phase while the other (spot 737) presented high abundance in the S and G2/M phases, indicating the different roles of this protein in the cell cycle progression of *P. donghaiense*. Overall, our results suggested that ABC transporters played essential roles in maintaining the proper progression of cell division in *P. donghaiense*cells.

Two proteins, methionine S-adenosyltransferase (SAM-S) and adenosylhomocysteinase (SAHH) are proposed to be involved in paralytic shellfish toxin (PST) synthesis in toxic dinoflagellate species [70]–[72]. It is reported that SAHH is down-regulated during toxin biosynthesis and the early G1 phase of cell cycle in *A. fundyense* [73], while SAM-S and SAHH expressions are up-regulated during PST production and the G2/M phase of cell cycle in *A. catenella* [72]. In addition, both genes are highly expressed at the transcriptomic level during cell proliferation [16]. Our study showed that SAM-S presented low abundance while SAHH exhibited high abundance in the G1 phase in *P. donghaiense*. These studies suggest that SAM-S and SAHH might play important roles in cell cycle regulation, but they are not unique to toxin-producing dinoflagellates, for they are also present in non-toxic species, i.e. *P. donghaiense*. The role of SAM and SAHH in toxin biosynthesis in dinoflagellates appears complex and needs further investigation.

It must be pointed out that no specific cyclin or CDK was identified in our study. The reason for this was not clear. Cyclins and CDks are presented at very low levels in cells and are subject to degradation [74], [75]. Our gel-based proteomic approach might not have been sensitive enough to detect cyclin or CDK proteins. Sampling time might have been another factor that affected the detection of cyclins and CDKs which are periodic expressing proteins. Moreover, the cyclin and CDK genes are scarcely represented in the present DinoEST [76]. In our study, there were a large number of proteins still unidentified due to the limitations of the genomic and proteomic dinoflagellate databases. These proteins might have belonged to the cyclin or CDK family. With an increasing genomic database of marine dinoflagellates and the application of new proteomic approaches (i.e. a shotgun proteomic approach), it is likely that we will gain more information about the molecular processes involved in cell cycle regulation on a proteomic scale, and this will certainly improve our understanding of the regulation of cell growth and proliferation of dinoflagellates. Moreover, comprehensive studies of protein patterns at different bloom stages and under different growth conditions will also provide new insights into the occurrence and decline of dinoflagellate blooms in the ocean.

## Conclusion

**Figure 6:**
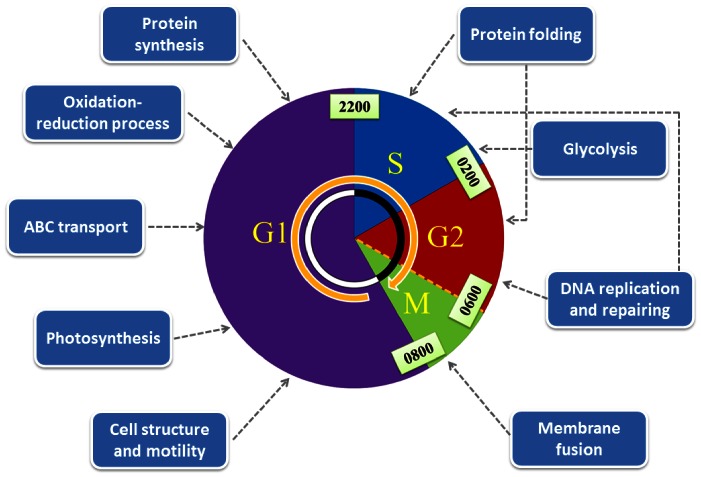
The proposed scheme illustrating essential cellular events occurring in the different cell cycle phases of *P.* donghaiense. In the G1 phase, biological processes related to cell structure and motility, protein synthesis, photosynthesis and oxidation-reduction occurred, while DNA synthesis, protein folding, glycolysis and membrane fusion took place in the S and G2/M phases.

This study, for the first time, compared the protein profiles of *P. donghaiense* at different cell cycle phases and identified differentially expressed proteins which varied with the cell cycle using the quantitative proteomic approach. The newly identified proteins were involved in the cell cycle and division machinery and in multiple regulatory processes supporting cellular dynamics. In the G1 phase, biological processes related to cell structure and motility, protein synthesis, photosynthesis and oxidation-reduction occurred, while DNA synthesis, protein folding, glycolysis and membrane fusion were active in the S and G2/M phases (Figure 6). A large number of proteins synthesized at a specific stage of the proliferating cycle suggested that periodic protein expression is critical for the cell either to guarantee the optimal utilization of resources or to maintain the proper order and function of the cell cycle. PCNA, DEAD/DEAH box helicase as well as those proteins involved in protein turnover and oxidative stress response processes played important roles in regulating the cell cycle progression of *P. donghaiense*. This study provided new insights into the mechanisms underlying cell growth and bloom formation of dinoflagellates.

## Materials and Methods

### Organism and Synchronization

The strain of *P. donghaiense* Lu was provided by the Culture Collection Center of Marine Bacteria and Algae of the State Key Laboratory of Marine Environmental Science, Xiamen University, China. This strain had been treated using antibiotics and no bacteria were detected in culture media. Unialgal isolates were routinely maintained in K culture medium [77] at 20°C under a 14∶10 h light:dark photoperiod at a light intensity of approximately 100 µmol photons m^−2^ s^−1^ provided by fluorescent lamps.

### Synchronization of *P. donghaiense*

Cultures of *P. donghaiense* were synchronized using a dark induced method. Synchronization of cells was achieved by maintaining the exponential growing cells in continuous darkness for 36 h. Subsequently, the vigorous synchronized cells were filtered using 10 µm filter meshes and rinsed three times using autoclaved seawater. The synchronized *P. donghaiense* cells were grown in glass tanks containing 60 L of K medium at 20°C under a light illumination of 100 µmol photon m^−2^ s^−1^ provided by cool-white fluorescent lamps on a 14∶10 h light: dark cycle. The light cycle was set from 0800 to 2200 h, and the dark cycle from 2200 to 0800 h. Samples for cell count, flow cytometric, proteome and western blotting analysis were taken every 2 h.

### Flow Cytometric Analysis

The cell cycle phases of *P. donghaiense* were determined by harvesting 60 mL of *P. donghaiens*e culture every two hours (n = 3) from 1600 to 1200 h throughout one diel cycle. All cultures of *P. donghaiense* were transferred to 50 mL centrifuge tubes, fixed with 70% ethanol and stored at 4°C for at least eighteen hours, then centrifuged (10 000×g for 3 min) to pellet the cells, and then washed twice with phosphate buffered saline (PBS). The permeabilized cells were again pelleted by centrifugation (10 000×g for 3 min), resuspended in PBS containing 10 mg mL^−1^ propidium iodide (PI, Sigma, St. Louis, MO) and 10 mg mL^−1^ RNase (Sigma, St. Louis, MO), and incubated in the dark for 1 hour at 37°C. DNA analysis of the PI stained cells was carried out on an Epics XL flow cytometer (Beckman Coulter, Miami, FL) using a 5 W argon laser with a 488 nm excitation wavelength and 635 nm emission wavelength. The cell cycle samples were analyzed using MultiCycle for Windows software with the same analysis model.

### Protein Extraction and Quantification

To compare differential protein expression, *P. donghaiense* cells were harvested at four time points, 0000, 0200, 0600 and 1200 h corresponding to the S, early G2/M, late G2/M, and G1 phases. For each time point, approximately 1×10^6^ cells of *P. donghaiense* were collected by centrifugation at 8 000×g for 30 min at 20°C. The pellet was subsequently transferred to a 1.5 mL microcentrifuge tube, rinsed twice with sterile seawater, and centrifuged again at 8000×g for 30 min at 20°C. 1 mL Trizol reagent was added to the cell pellet and it was subjected to sonication (a total of 2 min with short pulses of 5–10 s) on ice. Cell lysis was confirmed using light microscopy. Subsequently, 200 µL of chloroform was added to the cell lysate before shaking vigorously for 15 s. The mixture was allowed to stand for 5 min at room temperature before being centrifuged at 12 000×g for 15 min at 4°C. The top pale-yellow or colorless layer was removed, and then 300 µL of ethanol was added to resuspend the reddish bottom layer and the mixture centrifuged at 2000×g for 5 min at 4°C. The supernatant was transferred to a new tube and 1.5 mL of isopropanol added. The mixture was allowed to stand for at least 1 h for precipitation of proteins at room temperature. It was then centrifuged at 14000×g for 10 min at 4°C. The pellet obtained was briefly washed with 95% ethanol before being allowed to air dry. 100 µL of rehydration buffer containing 7 M urea (Bio-Rad), 2 M thiourea (Sigma), 2% CHAPS (Bio-Rad), 1% DTT (Bio-Rad), 0.5% immobilized pH gradient (IPG) buffer (GE Healthcare), and a trace of bromophenol blue (Bio-Rad) was added to solubilize the protein pellet. The solution was centrifuged at 20 000×g for 30 min at 16°C and the supernatant was collected for two-dimensional electrophoresis (2-DE) analysis. The protein content was quantified using a 2-D Quant kit (GE Healthcare, San Francisco, CA). Sample extraction and homogeneity were checked by visualization of silver staining proteins separated with 12% 2-DE.

### 2D-DIGE and Image Analysis

Protein samples were minimally labeled with Cy3 or Cy5 fluorescent dyes (40 µg of protein/320 pmol of dye) at 4°C following the manufacturer’s protocol and instructions (GE Healthcare). To minimize system and inherent biological variation, half of the samples from each cell cycle phase were labeled with Cy3, and the other half of the samples were labeled with Cy5. An internal standard was prepared by mixing equal amounts of all samples analyzed and was labeled with Cy2 fluorescent dye. Sample multiplexing was also randomizedto produce unbiased results (Table 2). IPG strips (pH 4–7, 24 cm, Bio-Rad) were loaded with 40 µg of each Cy2-, Cy3- and Cy5-labeled sample in rehydration buffer. Rehydration and subsequent isoelectric focusing were carried out in the Ettan IPGphor III Isoelectric Focusing System (GE Healthcare, Life Science) at 62 kV-h in different phases as follows: 6 h at 40 V, 6 h at 100 V, 30 min ramp up to 500 V, 1 h ramp up to 1000 V, 1 h ramp up to 2000 V, 1.5 h ramp up to 10,000 V, and 60 kv-h at 10,000 V.

**Table 2:** 2-D DIGE experimental design.

No. of gel	Cy2	Cy3	Cy5
1	Internal standard	S phase (1)	E G2/M phase (1)
2	Internal standard	L G2/M phase (1)	G1 phase (1)
3	Internal standard	S phase (2)	L G2/M phase (2)
4	Internal standard	G1 phase (2)	E G2/M phase (2)
5	Internal standard	G1 phase (3)	S phase (3)
6	Internal standard	E G2/M phase (3)	L G2/M phase (3)

After the first dimension was run, each strip was equilibrated with about 10 mL equilibration buffer containing 50 mM Tris (pH 8.8), 6 M urea, 30% glycerol, 2% SDS, 1% DTT, and a trace amount of bromophenol blue for 16 min. The strip was then placed in fresh equilibration buffer containing 2.5% iodoacetamide (instead of DTT) for another 16 min. Second dimension SDS-PAGE was run by overlaying the strips on 12% isocratic Laemmli gels (24×20 cm), which were cast in low fluorescence glass plates, on an Ettan DALT VI system. Gels were run at 10°C at a constant power of 1 watts/gel during 30 min followed by 15 watts/gel until the bromophenol blue tracking front had run off the gel. Fluorescent images of the gels were acquired on a Typhoon 9400 scanner (GE Healthcare). Cy2, Cy3, and Cy5 images for each gel were scanned at 488/520-, 532/580-, and 633/670-nm excitation/emission wavelengths, respectively, at 100-µm resolution, thus obtaining a total of 18 images (6×3).

Image analysis was performed using DeCyder version 7.0 software (GE Healthcare) following the manufacturer’s instructions. The differential in-gel analysis (DIA) module was used for intragel co-detection of samples and internal standard protein spots. Artifactual spots (dust and others) were filtered (maximum slope, <2.5; maximum peak height, <150) and removed. Analyses were done in initial spot detection number at 2500. The biological variation analysis (BVA) module was used for intergel matching of internal standard and samples across all gels and performing comparative cross-gel statistical analyses of all spots based on spot volumes, permitting the detection of differentially expressed spots between experimental conditions (ANOVA and Student’s t test, p<0.05) [30]. BVA was also done in initial spot number at 2500. Finally matches and the data quality of proteins of interest were manually checked to avoid false positives.

### Protein Identification

Preparative 2-D gels had a higher protein load (350 µg) for the four time points and were silver stained using the previously described method [30]. Owing to the selective staining properties of CyDye labeling and silver staining, 41 spots were visible and were excised from the silver-stained gels. Each set of spots was excised from replicate gels, destained twice with 200 mM ammonium bicarbonate in 50% acetonitrile/water for 20 min at 30°C, dehydrated with acetonitrileand then spun dried and in-gel digested with 10 ng/µL trypsin (Promega, UK) in 50 mMammonium bicarbonate overnight at 37°C overnight [78]. When needed, recovered peptides were desalted and concentrated with C18 ZipTips (Millipore), eluting peptides in 50% v/v ACN: water.

Samples were analyzed using an AB SCIEX MALDI TOF-TOF™ 5800 Analyzer (AB SCIEX, Shanghai, China) equipped with a neodymium: yttrium-aluminum-garnet laser (laser wavelength was 355 nm), using reflection positive ion mode. Protein identification was conducted according to the previously described method [31]. Briefly, with CHCA as the matrix, TFA for an ionization auxiliary reagent, and calibrated with Sequenzyme peptide standard kit (AB SCIEX), the MS spectra were processed using TOF/TOF Series Explorer software (AB SCIEX) allowing non-redundant and fully automated selection of precursors for MS/MS acquisition and data were acquired in the positive MS reflector mode with ascan range from 850 to 4000 Da. The MS and MS/MS spectra of each protein spot obtained from MALDI-TOF-TOF MS were first submitted to MASCOT search against the NCBI database with no taxonomic restriction (updated December, 2010, containing 4,607,655 entries). If the protein scores taken from the MS combined MS/MS search had a minimum C.I. of 95%, the protein hits were regarded as confident identifications. The other MS/MS spectra were subjected to similarity searches against the dinoflagellate EST database (updated March, 2011, containing 173,496 entries). The sequences were then subjected to similarity searches against the NCBI non-redundant protein database (nr) using the BLASTX algorithm [79]. If the total ion score C.I. was above 95% and the E value was below e^−20^ at the amino acid sequence level, the sequence similarities were considered to be significant. In the last, the rest unconfident hits were sequenced using *de novo* sequencing software (DeNovo Explorer™) to obtain candidate sequences and submitted to MS-BLAST searches. In the homology-based search, the statistical significance of hits was evaluated according to the MS BLAST scoring scheme. Only high-scoring segment pairs (HSSPs) with a score of 62 or above were considered to be confident.

### Immunoblot Analysis

To further validate the 2-D DIGE results, PCNA was selected for western blot analysis based on its higher fold difference on DIGE analysis and its potential role in cell cycle regulation. For each sample 25 µg protein was incubated at 95°C for 5 min and separated on 12.0% SDS-polyacrylamide gels (Hoefer SE 600 Ruby, GE Healthcare, USA) using the Laemmli buffer system. The proteins were transferred to a polyvinylidenedifluoride membrane (Millipore, Bedford, MA) using Trans-Blot SD Semi-Dry Transfer Cell (Bio-Rad, USA) at 200 mA for 3 h. The membrane was blocked with 5% non-fat dry milk for 2 h and then incubated with the primary antibody for 2 h at room temperature as follows: anti-PCNA (1∶1000 dilution; Xiamen University; Senjie Lin’s lab) and anti-tubulin (1∶1000 dilution; Abcam; Beyotime Institute of Biotechnology, Shanghai, China). Following three washes in PBS containing 0.05% Tween 20 (PBST), the membrane was incubated with a secondary anti-rabbit IgG (1∶1000 dilution; Beyotime Institute of Biotechnology, Shanghai, China) for 1 h at room temperature and then washed five times in PBST. Finally, the immunoblotting image was visualized using the Enhanced Chemiluminescent Substrate (Invitrogen) and the intensity of the bands was determined using densitometric analysis. The target protein signals were normalized to the tubulin signal and analyzed semiquantitatively using a Quantity One system. It should be pointed out that there are few dinoflagellate antibodies available for protein validation at present.

### Statistical Tests

Statistical analysis of protein expression levels was performed using two types of filters [80]. The first statistical filter was one-way ANOVA-1 (F-test, p≤0.05) to select the proteins that were regulated throughout the whole experiment with statistically significant changes. As the ANOVA test does not differentiate between groups and thus does not differentiate useful from useless comparisons, individual Student’s t-test comparisons and ratio calculations (p≤0.05) were also done between groups. The following comparisons were made: S phase versus G1 phase, early G2/M phase versus G1 phase, and finally late G2/M phase versus G1 phase.

## Supporting Information

Tables S1 **Includes Table S1 to S5.** Table S1. The general information of identified proteins in *P. donghaiense* cells at different cell cycle phases. Table S2. The information of identified proteins against the NCBI database using MASCOT search. Table S3. The information of identified proteins against the dinoflagellate EST database. Table S4. The information of identified proteins using de novo sequencing and MS-BLAST analysis. Table S5. The unidentified proteins with average relative change at each time point.
(XLS)
